# Symptom severity clusters in myeloproliferative neoplasms are unrelated to disease phenotype: results from a multicenter survey of the East German study group for hematology and oncology (OSHO #97)

**DOI:** 10.3389/fonc.2026.1802050

**Published:** 2026-03-23

**Authors:** Sabine Felser, Maximilian Koeppel, Rea Kuehl, Philipp le Coutre, Haifa K. Al-Ali, Susann Schulze, Lars-Olof Muegge, Julia Bormann, Jan Geissler, Armin Dadgar, Christian Junghanss, Sabina Ulbricht

**Affiliations:** 1 Department of Internal Medicine, Clinic for Hematology, Hemostaseology, Oncology, Stem Cell Therapy and Palliative Care, Rostock University Medical Center, Rostock, Germany; 2 Working Group Exercise Oncology, Department of Medical Oncology, Heidelberg University Hospital, Medical Faculty Heidelberg, Heidelberg University, National Center for Tumor Diseases Heidelberg, a partnership between DKFZ and Heidelberg University Hospital, Heidelberg, Germany; 3 Deutscher Verband für Gesundheitssport und Sporttherapie (DVGS) e.V., Hürt-Efferen, Germany; 4 Charité – Universitätsmedizin Berlin, Corporate Member of Freie Universität Berlin and Humboldt-Universität zu Berlin, and Berlin Institute of Health, Department of Hematology, Oncology, and Cancer Immunology, Berlin, Germany; 5 Krukenberg Cancer Center Halle (Saale), University Hospital Halle, Halle (Saale), Germany; 6 Department of Medicine Clinic II, Hematology, Oncology, Palliative Medicine, Carl-von-Basedow-Klinikum, Merseburg, Germany; 7 Department of Internal Medicine III, Heinrich Braun Klinikum Zwickau, Zwickau, Germany; 8 Leukaemie-Online/LeukaNET e.V, Riemering, Germany; 9 mpn-netzwerk e. V., Bonn, Germany; 10 Institut for Community Medicine, Department SHIP-KEF, University Medicine Greifswald, Greifswald, Germany

**Keywords:** chronic myeloid leukemia (CML), exercise therapy, fatigue, health-related quality of life (HRQoL), myeloproliferative neoplasms (MPN), supportive care

## Abstract

**Background:**

Myeloproliferative neoplasms (MPNs) comprise a heterogeneous group of clonal hematopoietic stem cell disorders with distinct molecular pathogenesis and treatment strategies. Across all MPN subtypes, patients experience a substantial and persistent symptom burden that impairs health-related quality of life (HrQoL). Because MPNs are typically chronic diseases, lifelong symptom management and supportive therapies play a central role in patient care. Supportive care primarily targets disease- and treatment-related symptoms rather than the underlying malignancy itself. However, it remains unclear whether symptom profiles are comparable across MPN subtypes independent of disease phenotype. Therefore, this analysis aimed to identify symptom severity clusters (SSCs) in a cohort of patients with MPN.

**Methods:**

A multicenter cross-sectional survey was conducted. Patients with MPN aged ≥18 years completed questionnaires assessing sociodemographic and disease-specific variables (e.g., sex, age, diagnosis, and current therapies) as well as the severity of 14 symptoms (e.g., fatigue, musculoskeletal pain, and itching), rated on continuous scales ranging from 0 (absent) to 100 (worst imaginable). Hierarchical and K-means cluster analysis as well as multinomial logistic regression were used to identify SSCs and their associations with patient characteristics.

**Results:**

The sample comprised 644 patients (63% female; mean age 56.8 ± 13.4 years), including 187 patients with chronic myeloid leukemia, 174 with polycythemia vera, 154 with essential thrombocythemia, and 129 with myelofibrosis. Four SSCs were identified: very high, high, middle, and low symptom severity. Compared with the low SSC, membership in the very high SSC was associated with younger age (95% CI: −0.04 to 0.00) and lower educational level (95% CI: −1.17 to −0.04). Membership in the high SSC was associated with female sex (95% CI: 1.26 to 2.60), higher body mass index (95% CI: 0.00 to 0.11), and lower educational level (95% CI: −1.20 to −0.19). Membership in the very high, high, or middle SSCs, compared with the low SSC, was not associated with diagnostic group.

**Conclusion:**

Symptom severity in patients with MPN appears to be comparable across disease subtypes. These findings support a shift toward symptom-focused, multimodal supportive care approaches (e.g., exercise and psychological support) in patients with MPN.

## 1 Introduction

Myeloproliferative neoplasms (MPN) comprise a heterogeneous group of clonal hematopoietic stem cell disorders that differ substantially in molecular pathogenesis and medical treatment strategies (1). Chronic myeloid leukemia (CML), defined by the presence of the *BCR::ABL1* fusion gene (Philadelphia chromosome–positive, Ph+), is primarily treated with tyrosine kinase inhibitors (TKIs), resulting in an excellent survival prognosis (2, 3). In contrast, Philadelphia chromosome–negative (Ph−) MPNs, including polycythemia vera (PV), essential thrombocythemia (ET), and myelofibrosis (MF), are managed using phlebotomy, cytoreductive agents such as interferon, Hydroxyurea or Janus kinase (JAK) inhibitors, depending on disease phenotype, symptom burden, and risk stratification. While patients with PV and ET often have a near-normal life expectancy, MF is associated with a markedly reduced survival, depending on individual risk stratification (4, 5).

Despite these fundamental differences in disease biology and treatment, patients across all MPN subtypes experience a substantial and often persistent symptom burden (6–10). Clinical manifestations range from mild or asymptomatic to highly symptomatic courses and are mainly characterized by fatigue, cognitive impairment, musculoskeletal pain, headache, dizziness, and impaired health-related quality of life (HrQoL). Furthermore, treatment-related adverse effects may contribute to and/or exacerbate this symptom burden. Given that MPN is usually a chronic disease, lifelong symptom management is a central component of comprehensive care (2, 5).

The objective of supportive therapies is threefold: first, to alleviate persistent and heterogeneous disease- and therapy-related symptoms; second, to maintain functionality; and third, to ensure long-term HrQoL. As such, they have become essential complementary measures in modern oncology (11). Exercise therapy is among the most extensively researched supportive interventions. International guidelines recommend structured exercise therapy as an integral part of oncological care (12). These recommendations are based on a substantial body of evidence demonstrating positive effects on symptoms such as fatigue, physical performance, and psychological well-being, as well as improvements in HrQoL, in both solid and hematological malignancies (13, 14). The primary focus of exercise therapy is on addressing symptoms associated with the disease and its treatment rather than the underlying malignancy itself (15). From this perspective, it seems reasonable to assume that patients with different diseases but comparable symptoms may benefit from similar exercise recommendations, regardless of molecular subtyping.

However, exercise-based research in hematology, particularly in MPNs, remains limited and unevenly distributed across disease entities. Most previous exercise intervention studies in MPN populations have focused almost exclusively on Ph− entities such as PV, ET, and MF, whereas patients with CML have been underrepresented (16–18). Although a number of hematological exercise studies have included small numbers of patients with CML, these patients have not been analyzed as a distinct subgroup (19). Research on exercise therapy in CML has so far been largely restricted to studies examining acute responses to exercise or physiological stress (20, 21). This unequal consideration of Ph+ and Ph− MPN patients in training studies suggests potentially different symptom burdens between the two groups, which could, in turn, imply different training needs.

However, initial results from the East German Study Group Hematology and Oncology (OSHO) #97 study appear to challenge this assumption. In that study, symptom profiles and symptom severity were analyzed in patients with CML and PV for the first time, and specific exercise recommendations were derived for each cohort (22, 23). Notably, both the incidence and severity of symptoms were comparable between the two cohorts, despite differences in disease biology and medical treatment strategies. Against this background, the aim of the present study was to analyze whether patients with Ph+ and Ph− MPN differ in terms of symptom severity, regardless of their underlying diagnosis. This question was addressed using a cluster analysis.

## 2 Methods

### 2.1 Study design, respondents and ethics

A cross-sectional study was conducted between January and September 2021 in 12 hospitals and collaborating institutions of OSHO. Patients aged ≥18 years with any type of MPN (24) were eligible for participation. Data were collected using a standardized paper-pencil questionnaire administered in clinical settings, as well as via an online platform for members of LeukaNET/Leukemia-Online and the German, Austrian, and Swiss MPN patient networks. The survey was approved by the Ethics Committee of the University of Rostock (A2020-0274) and registered in the German Registry of Clinical Trials (DRKS00023698).

### 2.2 Questionnaire

#### 2.2.1 General information

Data on sex, age, educational level, and partnership status were collected. Educational level was dichotomized into lower (≤10 years of schooling) and upper secondary education (>10 years of schooling). Body mass index (BMI) was calculated based on self-reported height and weight.

#### 2.2.2 Disease-related information

Participants were asked to report their MPN subtype, year of diagnosis, and current disease-specific therapies. TKIs, JAK inhibitors, oral chemotherapy (hydroxyurea), and interferon were grouped together under disease-specific pharmacological therapy.

#### 2.2.3 HrQoL and symptoms

HrQoL was assessed using a visual analogue scale (VAS) ranging from 0 (very poor) to 100 (very good). Fourteen frequently occurring symptoms were recorded using VASs ranging from 0 (absent) to 100 (worst imaginable). These included eight single items from the MPN Assessment Form (MPN-SAF) (25), supplemented by headache, dizziness, feeling of heat, nausea, vomiting, and diarrhea.

### 2.3 Statistical analysis

Data from 766 participants were transferred to a single electronic database. Statistical analyses were performed using SPSS version 25.0 (IBM Corp., Chicago, IL, USA) and Stata version 18 (StataCorp, College Station, TX, USA). A *p* value of <0.05 was considered statistically significant.

**Figure 1:**
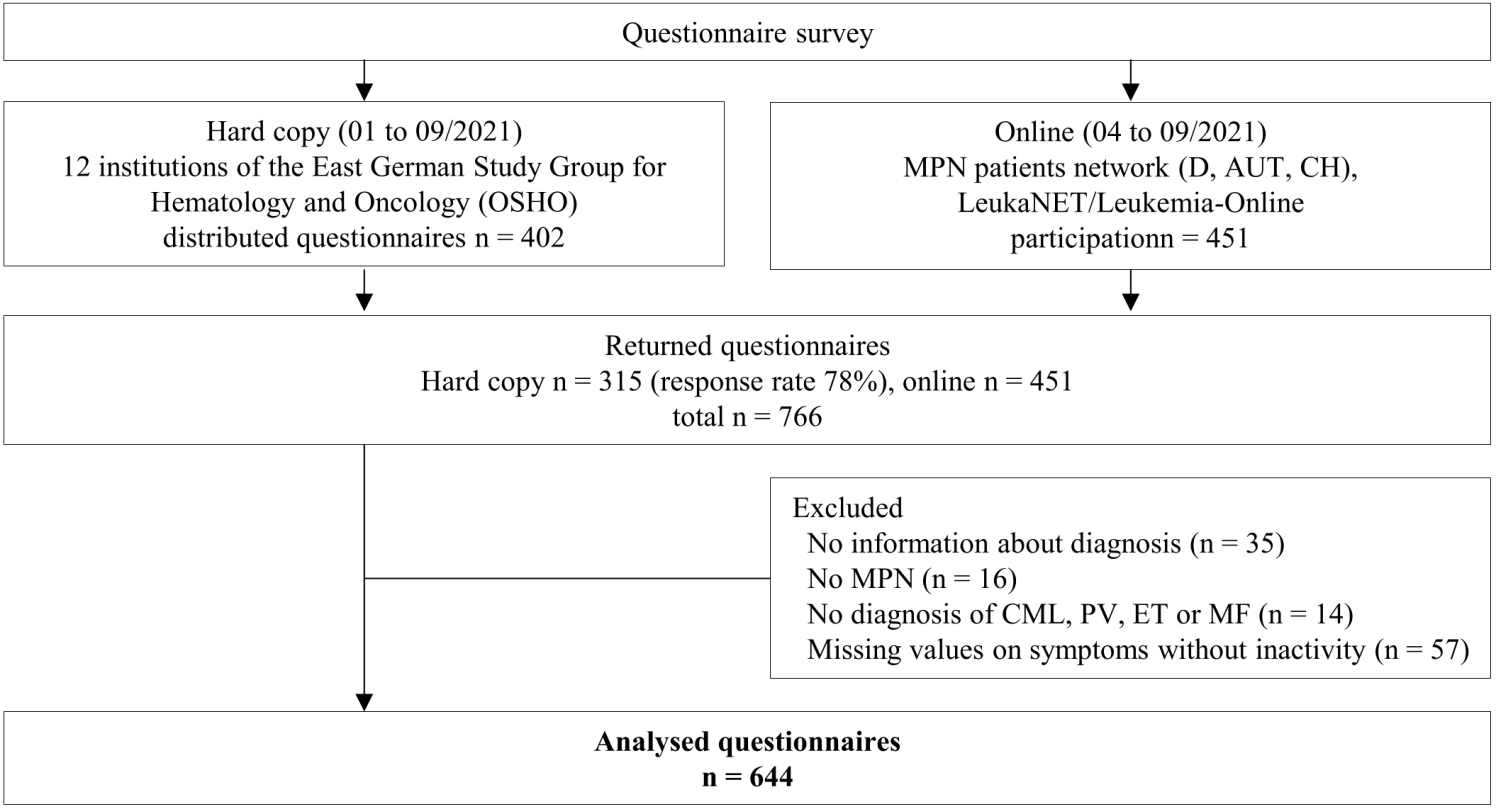
Flow chart of the study. D, Germany; AUT, Austria; CH, Switzerland, MPN, myeloproliferative neoplasms; CML, chronic myeloid leukemia; PV, polycythemia vera; ET, essential thrombocythemia; MF, myelofibrosis.

Records without information on a diagnosis of CML, PV, ET, or MF (including primary MF, post-PV MF, and post-ET MF), as well as records with incomplete symptom data, were excluded. This resulted in a final sample of 644 participants (**Figure 1**). Descriptive statistics are presented as mean ± standard deviation for numerical variables and as counts and percentages for categorical variables. Multicollinearity was assessed using Pearson’s correlation; variables with r >0.6 were excluded from further analyses (**Supplementary Table S1**).

**Figure 2:**
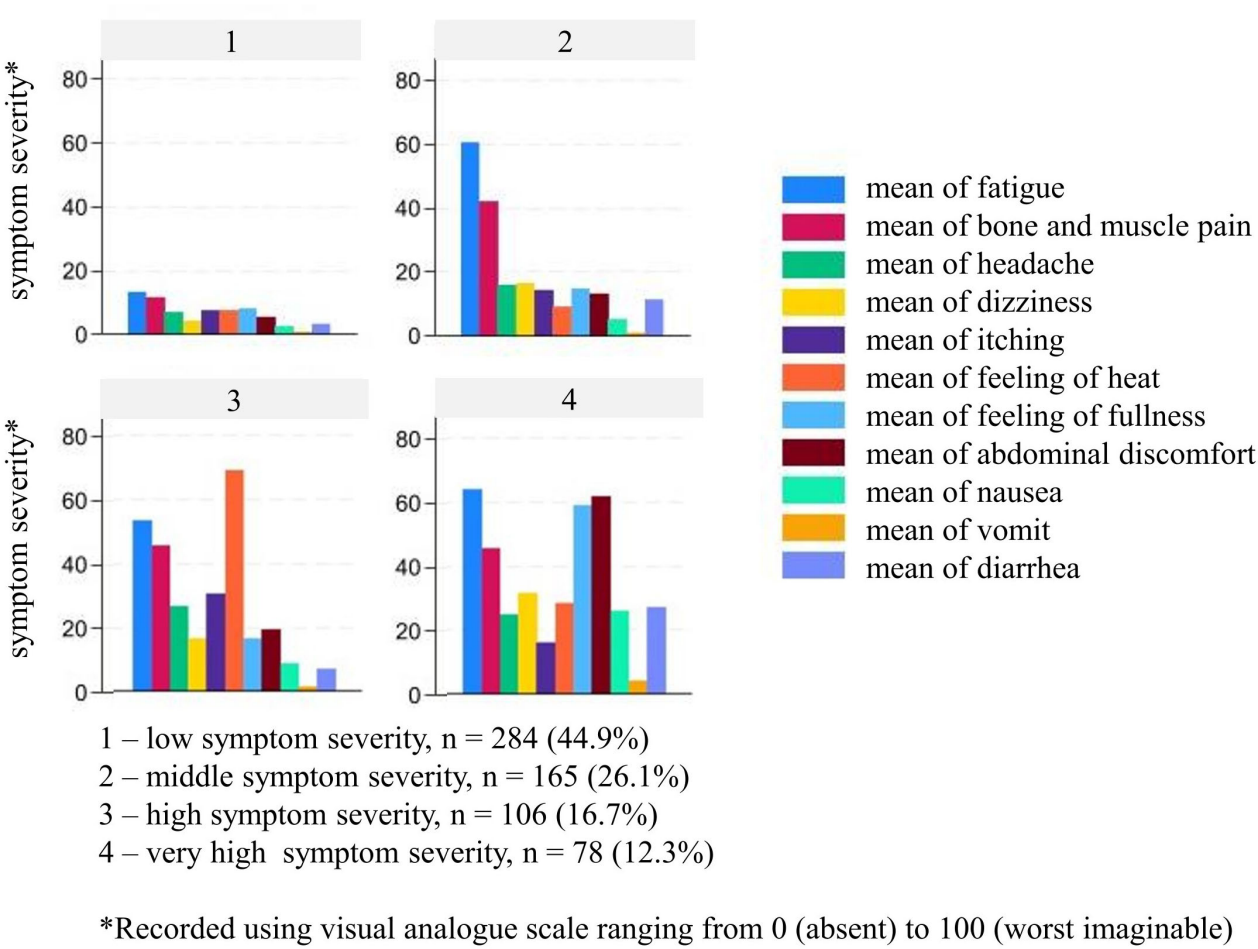
Symptom severity clusters (n = 633).

Hierarchical agglomerative cluster analysis was used to identify patient groups based on symptom severity. Firstly, the single-linkage algorithm, which recursively merges the two closest clusters based on minimal distance, was applied to detect outliers (26). Ten outliers were identified and excluded from further analyses. In addition, one patient with a BMI of >60 kg/m^2^ was excluded because of a suspected data entry error. Secondly, Ward’s linkage algorithm was applied to the remaining cases (n = 633) (27). The dendrogram and statistical criteria (Duda–Hart test) were evaluated to determine the optimal number of clusters, resulting in a four-cluster solution (**Supplementary Table S2**). Thirdly, the k-means algorithm was implemented (28). This is a widely used algorithm that classifies data into k distinct clusters based on a predetermined value. Clustering was performed utilizing the k-means method for the four clusters that had been identified previously through the hierarchical method. The process was expanded to encompass neighboring possible solutions with three or five clusters. For each setting, the silhouette width value (s_val) was calculated in order to assess the accuracy of the clustering assignment (29). The values of these parameters range from −1 to 1, with higher values indicating greater cohesion and separation. The optimal value for k was determined based on the highest average silhouette width value of the clustered data: k = 3 (s_val = 0.526), k = 4 (s_val = 0.545), and k = 5 (s_val = 0.516). The highest silhouette width value was obtained for k = 4. These four clusters represent patients with low, middle, high, and very high symptom severity. Symptom severity across clusters was visualized using bar charts (**Figure 2**). To examine patient characteristics associated with symptom severity cluster (SSC), a multinomial regression analysis was performed.

## 3 Results

### 3.1 Sample characteristics

The data set comprised 644 participants (63% female; mean age 57 ± 13 years), including 187 patients with CML, 174 with PV, 154 with ET, and 129 with MF. The MF subgroup included 94 patients with primary MF, 16 with post-PV MF, and 19 with post-ET MF. The patients had been diagnosed a mean of 7.3 ± 6.5 years prior to the survey. Of 644 patients, 633 reported their current therapy. Among 179 CML patients, 84% received TKIs and 10% were in the post-TKI phase; overall, 87% received disease-specific pharmacological therapy. This proportion was lower in PH− MPNs (PV 72%, ET 63%, MF 73%). In PV (n = 174), oral chemotherapy (33%), interferon (24%), and JAK inhibitors (21%) were most common; 39% reported phlebotomy and 40% anticoagulation. In ET (n = 152), oral chemotherapy (44%) predominated, followed by interferon (15%) and JAK inhibitors (7%); 24% were managed with watch-and-wait, and 55% received anticoagulation. Among MF patients (n = 128), 49% were treated with JAK inhibitors, 17% with oral chemotherapy, and 13% with interferon, while 19% were under watch-and-wait. The mean BMI was 25.7 ± 4.8 kg/m^2^ and was highest among patients with CML (26.3 ± 5.2 kg/m^2^). A lower educational level was reported by 42% of respondents, with proportions ranging from 39% to 46% depending on disease entity (**Supplementary Table S3**).

### 3.2 HrQoL and symptom severity

The mean HrQoL score among patients with MPN was 67.4 ± 21.5, with patients with CML reporting the highest scores and those with MF the lowest (71.5 ± 21.1 vs. 63.3 ± 21.5). Across the overall cohort and all four diagnostic groups, the highest symptom severity was reported for fatigue (39.0 ± 29.7), inactivity (30.9 ± 27.1), concentration problems (30.0 ± 27.2), and bone and muscle pain (29.7 ± 29.3) (**Supplementary Table S3**). All four symptoms were negatively correlated with HrQoL (r = −0.483 to −0.631; *p* < 0.001).

### 3.3 Correlations between symptoms

As illustrated by the correlation matrix, significant correlations were observed between most symptoms across all disease entities, with the exception of vomiting and diarrhea. Correlation coefficients greater than 0.6 were identified between fatigue, inactivity, and concentration problems, as well as between night sweats and feelings of heat. Consequently, inactivity, concentration problems, and night sweats were excluded from further analyses (**Supplementary Table S1**).

### 3.4 Symptom severity cluster

Patients in the low SSC (45%; n = 284) exhibited the lowest overall symptom burden. Patients in the middle SSC (26%; n = 165) reported pronounced fatigue and musculoskeletal pain, while other symptoms remained relatively low. The high SSC (17%; n = 196) comprised patients with generally high symptom severity, with feelings of heat emerging as the predominant symptom; fatigue and musculoskeletal pain were comparable to levels observed in the middle SSC. In this cluster, headache and itching were more pronounced than in the low and middle SSCs. The very high SSC represented the smallest group (12%; n = 78) and was characterized by the highest symptom severity. In addition to fatigue and musculoskeletal pain, gastrointestinal complaints were particularly prevalent, along with headache, dizziness, and feelings of heat. Nausea and diarrhea were more pronounced in this cluster than in the other three clusters (**Figure 2**).

Patient characteristics across SSCs are presented in **Table 1**. While the sex distribution was nearly balanced in the low SSC (54% female), women predominated in the high SSC (87%). Patients in the very high SSC were, on average, younger and more likely to have a lower educational level, similar to those in the high SSC and in contrast to patients in the low or middle SSCs.

**Table 1:** Characteristics of patients depending on the symptom severity cluster.

Charcteristica	Symptom severity cluster				Total cohort
	Low	Middle	High	Very high	
Number of patients	284 (45)	165 (26)	106 (17)	78 (12)	633 (100)
Sex, female	152 (39)	94 (24)	92 (23)	54 (14)	392 (100)
Proportion in the cluster [%]	54	57	87	69	62
Age [years]	56.4 ± 13.9	59.2 ± 13.5	57.3 ± 11.0	53.2 ± 12.6	56.9 ± 13.3
Body Mass Index [kg/m²]	25.3 ± 4.0	25.7 ± 4.8	26.3 ± 4.9	25.1 ± 5.2	25.6 ± 4.5
Lower secondary education	95 (37)	68 (27)	55 (22)	36 (14)	254 (100)
Proportion in the cluster [%]	33	41	52	46	40
Diagnosis					
CML	96 (52)	38 (21)	25 (14)	24 (13)	183 (100)
PV	65 (38)	48 (28)	39 (23)	21 (12)	173 (100)
ET	70 (46)	40 (26)	24 (16)	18 (12)	152 (100)
MF	53 (42)	39 (31)	18 (14)	15 (12)	125 (100)
Time after diagnosis [years]	7.0 ± 6.0	8.1 ± 6.4	6.8 ± 7.3	7.6 ± 7.5	7.3 ± 6.5
Pharmacological therapy^1^	197 (71)	128 (78)	79 (75)	60 (79)	472 (75)

Data are presented as the number of participants (%) for categorical variables and as mean ± standard deviation for continuous variables.

CML, chronic myeloid leukemia; PV, polycythemia vera; ET, essential thrombocythemia; MF, myelofibrosis;

(primary myelofibrosis, post polycythemia vera myelofibrosis, and post essential thrombocythemia myelofibrosis).

^1^Includes taking TKIs or JAK inhibitors, oral chemotherapy, and interferon.

Overall, 52% of patients with CML were assigned to the low SSC. This proportion was lower in the other diagnostic groups, ranging from 38% in PV to 46% in ET. However, the proportion of patients with CML in the high and very high SSCs was comparable to that observed in the other disease entities (**Table 1**).

### 3.5 Multinomial logistic regression

The low SSC served as the reference category. Compared with this group, patients in the very high SSC were younger (95% CI: −0.04 to 0.00; *p* = 0.023) and had a lower educational level (95% CI: −1.17 to −0.04; *p* = 0.035). Membership in the high SSC was associated with female sex (95% CI: 1.26 to 2.60; *p* < 0.001), lower educational level (95% CI: −1.20 to –0.19; *p* = 0.007), and higher BMI (95% CI: 0.00 to 0.11; *p* = 0.048). No association was observed between SSC membership and disease entity (**Table 2**). Excluding disease-specific pharmacological therapy from the analysis resulted in only marginal changes (**Supplementary Table S4**).

**Table 2:** Multinomial logistic regression with low symptom severity cluster as the base outcome (n = 566).

Cluster	Predictor	Coefficient	Standard error	z	*p*-value	95% Confidence interval
2 – middle symptom severity	Sex	0.17	0.22	0.76	0.447	-0.27 – 0.60
	Age	0.01	0.01	1.01	0.313	-0.01 – 0.03
	Body Mass Index	0.03	0.03	1.24	0.217	-0.02 – 0.08
	School education	-0.25	0.22	-1.12	0.262	-0.68 – 0.19
	Diagnosis					
	PV	0.39	0.32	1.20	0.231	-0.25 – 1.02
	ET	0.26	0.32	0.81	0.416	-0.36 – 0.88
	MF	0.40	0.33	1.20	0.231	-0.25 – 1.05
	Time after diagnosis	0.02	0.02	1.32	0.185	-0.01 – 0.05
	Pharmacological therapy^1^	0.46	0.25	1.83	0.067	-0.03 – 0.96
	_cons	-2.65	0.93	-2.86	0.004	-4.47 – -0.84
3 – high symptom severity	Sex	1.93	0.34	5.64	0.000**	1.26 – 2.60
	Age	0.01	0.01	0.54	0.592	-0.01 – 0.02
	Body Mass Index	0.05	0.03	1.98	0.048*	0.00 – 0.11
	School education	-0.70	0.26	-2.68	0.007*	-1.20 – 0.19
	Diagnosis					
	PV	0.36	0.36	1.00	0.317	-0.35 – 1.08
	ET	-0.45	0.39	-1.15	0.251	-1.22 – 0.32
	MF	-0.15	0.39	-0.39	0.700	-0.91 – 0.61
	Time after diagnosis	-0.02	0.02	-0.95	0.341	-0.07 – 0.02
	Pharmacological therapy^1^	-0.06	0.30	-0.18	0.854	-0.65 – 0.54
	_cons	-3.31	1.03	-3.23	0.001	-5.32 – -1.30
4 – very high symptom severity	Sex	0.54	0.31	1.74	0.081	-0.07 – 1.14
	Age	-0.02	0.01	-2.27	0.023*	-0.04 – 0.00
	Body Mass Index	-0.05	0.04	-1.40	0.163	-0.12 – 0.02
	School education	-0.61	0.29	-2.11	0.035*	-1.17 – -0.04
	Diagnosis					
	PV	0.35	0.40	0.89	0.376	-0.43 – 1.13
	ET	-0.02	0.42	-0.04	0.965	-0.85 – 0.81
	MF	0.34	0.43	0.80	0.425	-0.49 – 1.17
	Time after diagnosis	0.02	0.02	0.95	0.343	-0.02 – 0.07
	Pharmacological therapy^1^	0.47	0.34	1.38	0.166	0.20 – 1.14
	_cons	0.51	1.13	0.45	0.650	-1.70 – 2.73

PV, polycythemia vera; ET, essential thrombocythemia; MF, myelofibrosis; (primary myelofibrosis, post polycythemia vera myelofibrosis, and post essential thrombocythemia myelofibrosis).

^1^Includes taking TKIs or JAK inhibitors, oral chemotherapy, and interferon.

Bold: statistically significant, *p ≤0.05, **p ≤0.001.

## 4 Discussion

The aim of this study was to determine whether patients with Ph+ (CML) and those with Ph− (PV, ET, and MF) MPNs differ with respect to symptom severity and symptom profiles. Three main findings emerged. First, respondents could be allocated to four distinct SSCs. Second, the presence and severity of cancer-related symptoms were independent of MPN subtype. Third, patients with the highest symptom burden were more likely to be younger, be female, have a lower level of education, and have a higher BMI.

Consistent with previous studies, patients in our cohort reported a wide range of symptoms (6, 7, 9, 30). The highest mean symptom scores were observed for fatigue, concentration problems, musculoskeletal pain, and physical inactivity. Physical inactivity, in particular, is known to be a consequence of symptoms such as fatigue or pain (31).

Although 45% of patients exhibited a low symptom burden, more than half of the cohort (55%) experienced moderate, high, or very high symptom severity. To identify patterns in symptom distribution, a four-cluster solution was proposed. In clusters characterized by at least moderate symptom severity, fatigue and pain were similarly pronounced, underscoring their central role in MPN symptomatology and highlighting the need for targeted symptom management. The strong association between fatigue and concentration problems further suggests that cognitive impairment represents a related core symptom. Approximately one-third of patients with MPN reported additional severe symptoms beyond the typical manifestations of fatigue and pain.

One of the identified symptom severity clusters was characterized by high symptom burden, particularly hot flushes, which correlate with night sweats. Given the predominance of women (87%) and a median age of 57 years in this cluster, hormonal changes associated with the menopausal transition may have contributed to these symptoms and may substantially impair HrQoL (32). Up to 80% of women experience vasomotor symptoms during menopause, often persisting for several years (33, 34). It may therefore be hypothesized that MPN- and treatment-related symptoms coincide with menopausal complaints.

A proportion of 12% of patients was assigned to the very high SSC, representing the group with the highest overall symptom burden. These patients reported more frequent gastrointestinal symptoms (e.g., feeling of fullness, abdominal discomfort, diarrhea), and dizziness was also significantly more pronounced than in the middle and high SSCs. The most severe symptom burden was associated with younger age, female sex, and lower educational level, which has been consistently reported in both CML and Ph− MPNs (8, 30, 35, 36). Among patients with Ph− MPNs, women report higher overall symptom severity than men, particularly with respect to abdominal complaints and microvascular symptoms such as headache, fatigue, sleep disturbances, concentration problems, and dizziness, despite comparable risk scores, treatments, and comorbidities (35).

A key finding of this study is that SSCs were independent of MPN subtype. Across all disease entities, between 26% and 35% of patients experienced high or very high symptom burden. The proportion of patients receiving pharmacological therapy was comparable in all four SSCs, ranging between 71% and 79%. Longitudinal analyses have shown that symptom burden in patients with MPN does not substantially decrease over the course of the disease or during cytoreductive therapy (6, 8, 10). These findings indicate that a more pragmatic, symptom-oriented approach to care is required. One potential strategy is the integration of non-pharmacological interventions into existing treatment pathways. The need for supportive therapies is supported by well-established associations between high symptom burden and reduced HrQoL (37), impaired work productivity (37), and poorer survival (6, 38).

Non-pharmacological interventions should primarily target fatigue and pain because these symptoms predominate in three of the four SSCs, are strongly associated with, concentration problems, reduced HrQoL, and significantly limit physical activity (31). Current evidence suggests that exercise therapy and psychotherapy, either alone or in combination, are among the most promising approaches (16, 39–41). To enable patients with high symptom burden to benefit from these interventions at an early stage, treating physicians should routinely assess symptoms and inform patients about available supportive options. This is particularly relevant for younger and overweight patients, women, and those with lower educational levels.

The average BMI in our cohort exceeded recommended values, and higher BMI was associated with more severe symptoms, consistent with findings by Christensen et al. (42), who described a U-shaped relationship between BMI and symptom burden. Overweight and obesity, as well as physical inactivity, are associated with an increased risk of several health impairments, including diabetes mellitus, cardiovascular disease, and malignancies (43, 44). Accordingly, greater emphasis should be placed on weight management, supported by evidence linking higher BMI to increased vasomotor symptoms (45). Effective weight management requires multimodal behavioral strategies combining exercise and nutritional interventions (46). Weight reduction and adherence to general physical activity recommendations (at least 150 minutes of moderate-intensity aerobic exercise or 75 minutes of vigorous-intensity exercise combined with twice-weekly whole-body resistance training) (12, 47) may also mitigate the increased cardiovascular risk observed in patients with CML receiving long-term therapy with newer-generation tyrosine kinase inhibitors (3, 48). Similarly, reducing overweight and physical inactivity—key modifiers of thrombosis risk in patients with Ph− MPN—may improve overall survival (49).

### 4.1 Strengths and limitations

A major strength of this study is the inclusion of a large cohort of patients with MPN recruited from German-speaking countries, enhancing the robustness and generalizability of the findings within this healthcare context. However, selection bias due to the online survey cannot be ruled out. This is indicated in particular by the average interval of 7.6 years since diagnosis in MF patients. Even though median survival has improved significantly over the last decade, it is 84 months with a confidence interval of 70–95 months for the JAK inhibitor ruxolitinib (50). It can be assumed that some MF patients are in pre-fibrotic stages or fibrosis grade 1 and may remain clinically asymptomatic or oligosymptomatic, resulting in a symptom prevalence and severity comparable to that of other entities. This is supported by the fact that one-fifth of MF patients stated that they were under watch-and-wait. Symptom assessment was based on the MPN-SAF (25), a validated instrument widely used in clinical practice and trials for Ph− MPNs and known to correlate with HrQoL. To ensure applicability to Ph+ disease, the questionnaire was supplemented with CML-specific symptoms, allowing for comprehensive assessment across MPN entities. One limitation is that psychological symptoms such as anxiety, depression, and sadness were not systematically assessed. Existing evidence indicates that both patients with Ph− and Ph+ MPN have a higher prevalence of anxiety and depression than the non-cancer population (51). In Ph− MPN cohorts, anxiety prevalence is approximately 20% (52), with depressive symptoms ranging from 12% to 40%, depending on assessment method and treatment (52, 53). In CML, moderate to severe depressive symptoms have been observed in approximately 20% of patients (52, 53). As a result, it remains unclear whether inclusion of psychological symptoms would have altered the identified SSCs. Furthermore, information on pharmacological treatments was self-reported by patients and may therefore be subject to misclassification. To validate and refine our findings, future studies should incorporate additional clinical parameters, such as treatment duration, response status, and comorbidities. Recording menopausal or hormonal status would help determine whether vasomotor symptoms are disease-related or attributable to other causes. In addition, modifiable lifestyle factors, including tobacco and alcohol consumption, should also be assessed, as they may influence symptom burden. Inclusion of patients from diverse ethnic backgrounds is necessary to improve the generalizability of our results and to support the development of a clinically applicable screening algorithm.

## 5 Conclusion and implications

This study identified four diagnosis-independent SSCs in patients with Ph+ and Ph− MPNs, demonstrating that symptom burden is primarily driven by patient characteristics rather than disease biology or treatment. Fatigue and pain emerged as dominant symptoms in three of the four clusters and were closely associated with concentration problems, physical inactivity and reduced HrQoL. These findings support a shift toward symptom-focused, multimodal supportive care approaches integrating exercise therapy, psychological support, and nutritional counseling.

Future research should examine how regular symptom assessment and tailored non-pharmacological interventions can be effectively implemented in routine clinical care and whether their benefits are consistent across both Ph+ and Ph− MPN entities. Larger international studies involving patients of different ethnicities are needed to potentially derive a clinical screening algorithm.

## Data Availability

The datasets presented in this study can be found in online repositories. The names of the repository/repositories and accession number(s) can be found below: The raw data supporting the conclusions of this article will be made available by the authors without undue reservation. The generated and analyzed datasets are available in the NFDI4Health repository: https://ldh.mediz-rostock.imise.uni-leipzig.de/projects/9#datafiles.
