# Correction: Symptom severity clusters in myeloproliferative neoplasms are unrelated to disease phenotype: results from a multicenter survey of the East German study group for hematology and oncology (OSHO #97)

**DOI:** 10.3389/fonc.2026.1837022

**Published:** 2026-04-06

**Authors:** Sabine Felser, Maximilian Koeppel, Rea Kuehl, Philipp le Coutre, Haifa K. Al-Ali, Susann Schulze, Lars-Olof Muegge, Julia Bormann, Jan Geissler, Armin Dadgar, Christian Junghanss, Sabina Ulbricht

**Affiliations:** 1 Department of Internal Medicine, Clinic for Hematology, Hemostaseology, Oncology, Stem Cell Therapy and Palliative Care, Rostock University Medical Center, Rostock, Germany; 2 Working Group Exercise Oncology, Department of Medical Oncology, Heidelberg University Hospital, Medical Faculty Heidelberg, Heidelberg University, National Center for Tumor Diseases Heidelberg, a partnership between DKFZ and Heidelberg University Hospital, Heidelberg, Germany; 3 Deutscher Verband für Gesundheitssport und Sporttherapie (DVGS) e.V., Hürt-Efferen, Germany; 4 Charité – Universitätsmedizin Berlin, Corporate Member of Freie Universität Berlin and Humboldt-Universität zu Berlin, and Berlin Institute of Health, Department of Hematology, Oncology, and Cancer Immunology, Berlin, Germany; 5 Krukenberg Cancer Center Halle (Saale), University Hospital Halle, Halle (Saale), Germany; 6 Department of Medicine Clinic II, Hematology, Oncology, Palliative Medicine, Carl-von-Basedow-Klinikum, Merseburg, Germany; 7 Department of Internal Medicine III, Heinrich Braun Klinikum Zwickau, Zwickau, Germany; 8 Leukaemie-Online/LeukaNET e.V., Riemering, Germany; 9 mpn-netzwerk eV., Bonn, Germany; 10 Institut for Community Medicine, Department SHIP-KEF, University Medicine Greifswald, Greifswald, Germany

**Keywords:** chronic myeloid leukemia (CML), exercise therapy, fatigue, health-related quality of life (HRQoL), myeloproliferative neoplasms (MPN), supportive care

Affiliation “Working Group Exercise Oncology, Department of Medical Oncology, Heidelberg University Hospital, Medical Faculty Heidelberg, Heidelberg University, National Center for Tumor Diseases Heidelberg, a partnership between DKFZ and Heidelberg University Hospital, Heidelberg, Germany” was erroneously given as “Department of Medical Oncology, Heidelberg University Hospital, Medical Faculty Heidelberg, Heidelberg University, Heidelberg, Germany” and “National Center for Tumor Diseases Heidelberg, A Partnership Between DKFZ and Heidelberg University Hospital, Heidelberg, Germany”. Affiliation “Charité – Universitätsmedizin Berlin, Corporate Member of Freie Universität Berlin and Humboldt-Universität zu Berlin, and Berlin Institute of Health, Department of Hematology, Oncology, and Cancer Immunology, Berlin, Germany” was erroneously given as “Medical Clinic with emphasis on Hematology and Oncology, Charité, Berlin, Germany”.

Affiliation “mpn-netzwerk e. V., Bonn, Germany” was erroneously given as “mpn-netzwerk e. V., Greifswald, Germany”

Affiliation “Institut for Community Medicine, Department SHIP-KEF, University Medicine Greifswald, Greifswald, Germany” was erroneously given as “Institut for Community Medicine, Department SHIP-KEF, University Medicine Greifswald, Bonn, Germany”.

There was a mistake in the published **Table 1, 2**

**Table 1**. “Diagnosis” is highlighted in gray and displayed in a larger font size than the other characteristics. In the corrected **Table 1**, “Diagnosis” should have a white background and the same font size as the other characteristics.

There are two mistakes in **Table 2**: First, column 1 reads “2 – middle symptom severitycluster” and “3 – high symptom severity cluster.” Since the column heading is “Cluster” the word “Cluster” should be removed from both entries. The correct designation is “2 – middle symptom severity” and “3 – high symptom severity”. Second, in row ET, column “Standard error (2 - middle symptom severity),” the value is 0. 32. The correct value in **Table 2** is: 0.32.

The original version of this article has been updated.

