# Supplementary material for: Symptom severity clusters in myeloproliferative neoplasms are unrelated to disease phenotype: results from a multicenter survey of the East German study group for hematology and oncology (OSHO #97)
Source: Front Oncol. 2026 Mar 23;16:1802050. doi: 10.3389/fonc.2026.1802050 (PMC13050751; doi:10.3389/fonc.2026.1802050)
Supplement: Supplementary file 1 [file DataSheet1.pdf]

S1. Pearson-Correlation (2-sided)

| | | | 1 | 2 | 3 | 4 | 5 | 6 | 7 | 8 | 9 | 10 | 11 | 12 | 13 | 14 |
|---|---|---|---|---|---|---|---|---|---|---|---|---|---|---|---|---|
| 1 | Fatigue | r | 1 | | | | | | | | | | | | | |
| | | *p*-value | | | | | | | | | | | | | | |
| | | N | 644 | | | | | | | | | | | | | |
| 2 | Inactivity | r | ,735** | 1 | | | | | | | | | | | | |
| | | *p*-value | 0,000 | | | | | | | | | | | | | |
| | | N | 630 | 630 | | | | | | | | | | | | |
| 3 | Concentration problems | r | ,626** | ,612** | 1 | | | | | | | | | | | |
| | | *p*-value | 0,000 | 0,000 | | | | | | | | | | | | |
| | | N | 644 | 630 | 644 | | | | | | | | | | | |
| 4 | Bone and muscle pain | r | ,464** | ,440** | ,369** | 1 | | | | | | | | | | |
| | | *p*-value | 0,000 | 0,000 | 0,000 | | | | | | | | | | | |
| | | N | 644 | 630 | 644 | 644 | | | | | | | | | | |
| 5 | Headache | r | ,359** | ,356** | ,320** | ,303** | 1 | | | | | | | | | |
| | | *p*-value | 0,000 | 0,000 | 0,000 | 0,000 | | | | | | | | | | |
| | | N | 644 | 630 | 644 | 644 | 644 | | | | | | | | | |
| 6 | Dizziness | r | ,384** | ,378** | ,379** | ,263** | ,341** | 1 | | | | | | | | |
| | | *p*-value | 0,000 | 0,000 | 0,000 | 0,000 | 0,000 | | | | | | | | | |
| | | N | 644 | 630 | 644 | 644 | 644 | 644 | | | | | | | | |
| 7 | Itching | r | ,213** | ,185** | ,159** | ,212** | ,168** | ,111** | 1 | | | | | | | |
| | | *p*-value | 0,000 | 0,000 | 0,000 | 0,000 | 0,000 | 0,005 | | | | | | | | |
| | | N | 644 | 630 | 644 | 644 | 644 | 644 | 644 | | | | | | | |
| 8 | Feeling of heat | r | ,311** | ,290** | ,328** | ,290** | ,259** | ,202** | ,290** | 1 | | | | | | |
| | | *p*-value | 0,000 | 0,000 | 0,000 | 0,000 | 0,000 | 0,000 | 0,000 | | | | | | | |
| | | N | 644 | 630 | 644 | 644 | 644 | 644 | 644 | 644 | | | | | | |
| 9 | Feeling of fullness | r | ,308** | ,295** | ,299** | ,241** | ,189** | ,280** | ,106** | ,213** | 1 | | | | | |
| | | *p*-value | 0,000 | 0,000 | 0,000 | 0,000 | 0,000 | 0,000 | 0,007 | 0,000 | | | | | | |
| | | N | 644 | 630 | 644 | 644 | 644 | 644 | 644 | 644 | 644 | | | | | |
| 10 | Abdominal discomfort | r | ,353** | ,337** | ,305** | ,278** | ,258** | ,245** | ,153** | ,208** | ,533** | 1 | | | | |
| | | *p*-value | 0,000 | 0,000 | 0,000 | 0,000 | 0,000 | 0,000 | 0,000 | 0,000 | 0,000 | | | | | |
| | | N | 644 | 630 | 644 | 644 | 644 | 644 | 644 | 644 | 644 | 644 | | | | |
| 11 | Nausea | r | ,252** | ,222** | ,243** | ,201** | ,207** | ,318** | ,175** | ,229** | ,439** | ,474** | 1 | | | |
| | | *p*-value | 0,000 | 0,000 | 0,000 | 0,000 | 0,000 | 0,000 | 0,000 | 0,000 | 0,000 | 0,000 | | | | |
| | | N | 644 | 630 | 644 | 644 | 644 | 644 | 644 | 644 | 644 | 644 | 644 | | | |
| 12 | Vomit | r | 0,050 | ,129** | 0,070 | ,152** | ,153** | ,164** | ,097* | ,116** | ,164** | ,159** | ,359** | 1 | | |
| | | *p*-value | 0,201 | 0,001 | 0,078 | 0,000 | 0,000 | 0,000 | 0,014 | 0,003 | 0,000 | 0,000 | 0,000 | | | |
| | | N | 644 | 630 | 644 | 644 | 644 | 644 | 644 | 644 | 644 | 644 | 644 | 644 | | |
| 13 | Diarrhea | r | ,202** | ,224** | ,206** | ,140** | 0,009 | ,192** | 0,025 | 0,064 | ,243** | ,365** | ,199** | ,194** | 1 | |
| | | *p*-value | 0,000 | 0,000 | 0,000 | 0,000 | 0,815 | 0,000 | 0,526 | 0,105 | 0,000 | 0,000 | 0,000 | 0,000 | | |
| | | N | 644 | 630 | 644 | 644 | 644 | 644 | 644 | 644 | 644 | 644 | 644 | 644 | 644 | |
| 14 | Night sweats | r | ,291** | ,259** | ,268** | ,248** | ,177** | ,153** | ,234** | ,625** | ,227** | ,179** | ,166** | ,103** | ,091* | 1 |
| | | *p*-value | 0,000 | 0,000 | 0,000 | 0,000 | 0,000 | 0,000 | 0,000 | 0,000 | 0,000 | 0,000 | 0,000 | 0,009 | 0,020 | |
| | | N | 644 | 630 | 644 | 644 | 644 | 644 | 644 | 644 | 644 | 644 | 644 | 644 | 644 | 644 |

red, removed from analysis; *p ≤0,05; **p ≤0,01
