# Supplementary material for: Symptom severity clusters in myeloproliferative neoplasms are unrelated to disease phenotype: results from a multicenter survey of the East German study group for hematology and oncology (OSHO #97)
Source: Front Oncol. 2026 Mar 23;16:1802050. doi: 10.3389/fonc.2026.1802050 (PMC13050751; doi:10.3389/fonc.2026.1802050)
Supplement: Supplementary file 2 [file DataSheet2.pdf]

S2. Results of Single Link and Ward Link cluster analysis

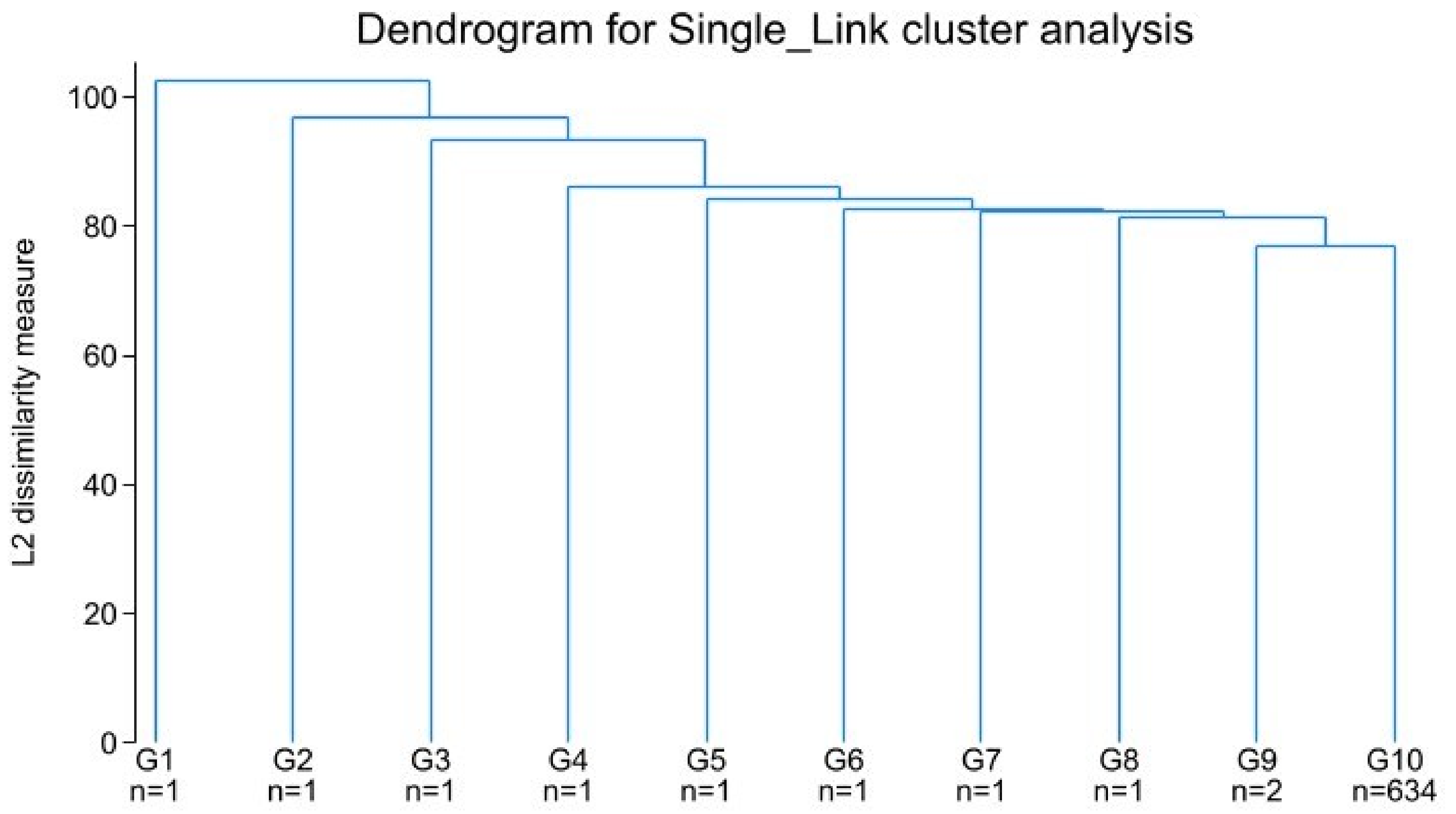

Single_10Linkage	Freq.	Percent	Cum.
1	1	0.16	0.16
2	1	0.16	0.31
3	1	0.16	0.47
4	1	0.16	0.62
5	1	0.16	0.78
6	1	0.16	0.93
7	1	0.16	1.09
8	1	0.16	1.24
9	2	0.31	1.55
10	634	98.45	100.00
Total	644	100.00	

G1 to G9 removed from analysis (outlier)

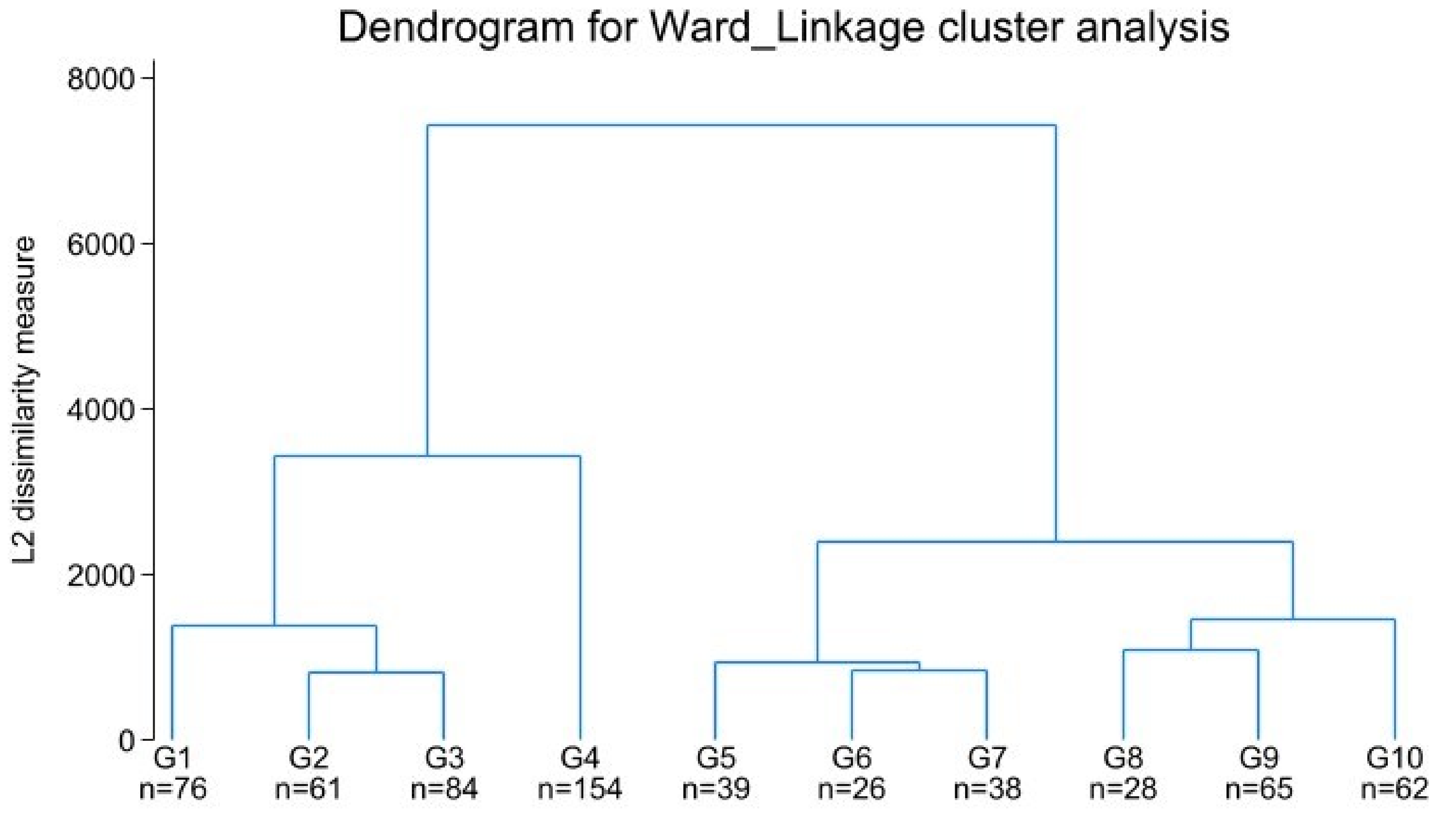

Number of clusters	Duda/Hart Je(2)/Je(1)	pseudo T-squared
1	0.8096	148.44
2	0.8045	90.65
3	0.8583	42.26
4	0.8457	27.92
5	0.8484	39.14
6	0.8022	22.44
7	0.8680	15.35
8	0.7999	15.51
9	0.8738	20.64
10	0.7309	27.24
