# Supplementary material for: Symptom severity clusters in myeloproliferative neoplasms are unrelated to disease phenotype: results from a multicenter survey of the East German study group for hematology and oncology (OSHO #97)
Source: Front Oncol. 2026 Mar 23;16:1802050. doi: 10.3389/fonc.2026.1802050 (PMC13050751; doi:10.3389/fonc.2026.1802050)
Supplement: Supplementary file 3 [file DataSheet3.pdf]

S3. Characteristics of patients with myeloproliferative neoplasms depending on the diagnosis

	Total cohort	CML	PV	ET	MF[a]
Total sample size	644	187	174	154	129
**Demographics**					
Sex, female	403 (63)	98 (53)	118 (68)	113 (73)	74 (57)
Age [years]	56.8 ± 13.4	51.5 ± 13.3	60.5 ± 12.1	55.8 ± 14.3	60.6 ± 11.3
Body Mass Index [kg/m²]	25.7 ± 4.8	26.3 ± 5.2	25.1 ± 4.5	25.1 ± 4.1	26.2 ± 5.2
School education, lower secondary education	261 (42)	69 (39)	71 (42)	68 (46)	53 (42)
Profession, working	354 (56)	123 (67)	87 (51)	85 (56)	59 (46)
Time after diagnosis [years]	7.3 ± 6.5	7.2 ± 6.0	8.0 ± 7.6	6.4 ± 5.8	7.6 ± 6.1
**Current therapies, n**	**633**	**179**	**174**	**152**	**128**
Phlebotomy	86 (14)	1 (1)	69 (39)	9 (6)	7 (6)
Anticoagulation	192 (30)	5 (3)	70 (40)	84 (55)	33 (26)
Transfusion erythrocyte concentrates	11 (2)	2 (1)	1 (1)	0 (0)	8 (6)
Tyrosine kinase inhibitor, TKI	150 (24)	150 (84)	-	-	-
Januskinase inhibitors	109 (17)	-	37 (21)	10 (7)	62 (49)
Cytostatics	150 (24)	5 (3)	57 (33)	66 (44)	22 (17)
Interferon	86 (14)	6 (3)	42 (24)	22 (15)	16 (13)
Stem cell transplantation	12 (2)	7 (4)	0 (0)	0 (0)	5 (4)
Post-TKI, watch-and-wait	90 (14)	18 (10)	12 (7)	36 (24)	24 (19)
Health-related quality of life[1]	67.4 ± 21.5	71.5 ± 21.1	64.3 ± 21.4	69.5 ± 21.0	63.3 ± 21.5
**Symptoms[2]**					
Fatigue	39.0 ± 29.7	35.9 ± 29.6	43.3 ± 28.9	35.8 ± 29.8	41.3 ± 30.1
Inactivity	30.9 ± 27.1	27.7 ± 27.4	34.6 ± 27.0	27.4 ± 25.4	34.8 ± 28.2
Concentration problems	30.0 ± 27.2	27.0 ± 26.0	32.7 ± 27.5	29.6 ± 27.2	31.1 ± 28.2
Bone and muscle pain	29.7 ± 29.3	28.5 ± 28.8	29.3 ± 28.6	28.1 ± 29.4	34.1 ± 30.5
Headache	15.1 ± 22.1	14.0 ± 23.3	13.8 ± 20.6	17.8 ± 23.1	15.3 ± 20.9
Dizziness	13.2 ± 20.4	11.9 ± 19.9	15.7 ± 21.7	13.5 ± 21.1	11.3 ± 18.4
Itching	14.6 ± 23.1	11.8 ± 21.1	21.1 ± 25.9	13.8 ± 23.1	10.9 ± 20.3
Feeling of heat	21.3 ± 28.3	16.6 ± 25.0	26.7 ± 29.5	21.8 ± 29.1	20.3 ± 29.1

Feeling of fullness	18.1 ± 24.5	16.9 ± 25.0	18.8 ± 24.1	16.2 ± 25.1	23.1 ± 25.1
Abdominal discomfort	17.4 ± 24.9	17.4 ± 25.8	17.8 ± 23.8	16.8 ± 25.8	21.3 ± 23.6
Nausea	7.8 ± 16.4	7.8 ± 16.1	7.9 ± 16.1	9.7 ± 18.5	5.1 ± 14.1
Vomit	1.6 ± 7.4	3.0 ± 11.5	0.9 ± 3.3	1.9 ± 6.9	0.4 ± 1.2
Diarrhea	9.3 ± 19.1	12.7 ± 23.3	7.8 ± 18.1	6.3 ± 13.3	9.9 ± 19.0
Night sweats	19.0 ± 27.1	16.0 ± 25.9	23.6 ± 28.8	18.0 ± 26.7	18.2 ± 26.5

Data are presented as the number of participants (%) for categorical variables and as mean ± standard deviation for continuous variables.
Abbreviations: *CML*, chronic myeloid leukemia; *PV*, polycythemia vera; *ET*, essential thrombocythemia; *MF*, myelofibrosis;
[a]primary myelofibrois (n = 94), post polycythemia vera myelofibrosis (n = 16), and post essential thrombocythemia myelofibrosis (n = 19)
[1]range 0 to 100, higher values represent high health-related quality of life; [2]range 0 to 100, higher values represent more discomfort.
