# Supplementary material for: Symptom severity clusters in myeloproliferative neoplasms are unrelated to disease phenotype: results from a multicenter survey of the East German study group for hematology and oncology (OSHO #97)
Source: Front Oncol. 2026 Mar 23;16:1802050. doi: 10.3389/fonc.2026.1802050 (PMC13050751; doi:10.3389/fonc.2026.1802050)
Supplement: Supplementary file 4 [file DataSheet4.pdf]

S4. Multinomial logistic regression with low symptom severity cluster as the base outcome (n = 570)

	Predictor	Coefficient	Standard error	z	*p*-value	95% confidence interval
2 – middle symptom severity	Sex	0.20	0.22	0.89	0.371	-0.24 – 0.63
	Age	0.01	0.01	1.13	0.259	-0.01 – 0.03
	Body Mass Index	0.03	0.03	1.07	0.283	-0.02 – 0.08
	School education	-0.24	0.22	-1.07	0.284	-0.67 – 0.20
	Diagnosis					
	PV	0.35	0.32	1.11	0.267	-0.27 – 0.97
	ET	0.19	0. 31	0.61	0.541	-0.42 – 0.79
	MF	0.36	0.32	1.11	0.269	-0.28 – 0.99
	Time after diagnosis	0.02	0.02	1.55	0.120	-0.01 – 0.05
	_cons	-2.22	0.91	-2.46	0.014	-4.00 – -0.45
3 – high symptom severity cluster	Sex	1.82	0.32	5.63	**0.000****	1.19 – 2.45
	Age	0.00	0.01	0.43	0.666	-0.01 – 0.02
	Body Mass Index	0.05	0.03	1.96	**0.049***	0.00 – 0.11
	School education	-0.66	0.26	-2.57	**0.010***	-1.16 – -0.16
	Diagnosis					
	PV	0.417	0.35	1.19	0.234	-0.27 – 1.10
	ET	-0.37	0.37	-1.02	0.308	-1.09 – 0.35
	MF	-0.11	0.38	-0.28	0.779	-0.85 – 0.64
	Time after diagnosis	-0.02	0.02	0.86	0.391	-0.06 – 0.03
	_cons	-3.34	1.00	-3.34	0.001	-5.30 – -1.38
4 – very high symptom severity	Sex	0.57	0.31	1.86	0.063	-0.03 – 1.17
	Age	-0.02	0.01	-2.16	**0.031***	-0.04 – 0.00
	Body Mass Index	-0.05	0.03	-1.51	0.130	-0.12 – 0.02
	School education	-0.60	0.29	-2.09	**0.037***	-1.16 – -0.04
	Diagnosis					
	PV	0.29	0.40	0.74	0.462	-0.48 – 1.07
	ET	-0.12	0.42	-0.29	0.768	-0.94 – 0.69
	MF	0.28	0.42	0.68	0.494	-0.53 – 1.10
	Time after diagnosis	0.02	0.02	1.06	0.287	-0.02 – 0.07
	_cons	0.96	1.06	0.91	0.365	-1.12 – 3.05

Abbreviations: *PV*, polycythemia vera; *ET*, essential thrombocythemia; *MF*, myelofibrosis;
(primary myelofibrois, post polycythemia vera myelofibrosis, and post essential thrombocythemia myelofibrosis)
Bold: statistically significant, * $p \leq 0.05$, ** $p \leq 0.001$
